# Monkeypox Outbreak — Nine States, May 2022

**DOI:** 10.15585/mmwr.mm7123e1

**Published:** 2022-06-10

**Authors:** Faisal S. Minhaj, Yasmin P. Ogale, Florence Whitehill, Jordan Schultz, Mary Foote, Whitni Davidson, Christine M. Hughes, Kimberly Wilkins, Laura Bachmann, Ryan Chatelain, Marisa A.P. Donnelly, Rafael Mendoza, Barbara L. Downes, Mellisa Roskosky, Meghan Barnes, Glen R. Gallagher, Nesli Basgoz, Victoria Ruiz, Nang Thu Thu Kyaw, Amanda Feldpausch, Amy Valderrama, Francisco Alvarado-Ramy, Chad H. Dowell, Catherine C. Chow, Yu Li, Laura Quilter, John Brooks, Demetre C. Daskalakis, R. Paul McClung, Brett W. Petersen, Inger Damon, Christina Hutson, Jennifer McQuiston, Agam K. Rao, Ermias Belay, Andrea M. McCollum, Monkeypox Response Team 2022, Monkeypox Response Team 2022, Kristina Angelo, Matt Arduino, Ray Arthur, Nicolle Baird, Jonathan Batross, Amy Beeson, Jui Bhingarde, Michael Bowen, Clive Brown, Catherine M. Brown, Alexis Burakoff, Kelly Charniga, Tai-Ho Chen, Sherry Chen, Patrick Clay, James Cope, Jennifer Cope, Michelle Addo Dankwa, Lisa Delaney, Marie De Perio, Michelle Decenteceo, Kristin Delea, Jeffrey B. Doty, Jeffrey Duchin, Joseph Dunlap, Ryan Fagan, Bryce Furness, Shannon Gearhart, Crystal Gigante, Aubrey Gilliland, Lucas Gosdin, Isabel Griffin, Amanda Groccia, Sarah Guagliardo, Yonette Hercules, Kelly Jackson, Paulino Jarquin, Rachel Kachur, Alexander Kallen, Raymond Kao, Aubree Kelly, Mohammed Khan, Theodora Khan, Aaron Kofman, Krista Kornylo, David Kuhar, Michael LaFlam, Robert Lash, Andrew Lashombe, David Lowe, Amanda MacGurn, Nina Masters, Keegan McCaffrey, Jenna L. Mink, Benjamin Monroe, Clint N. Morgan, Yoshinori Nakazawa, Julie Nash, Terese Navarra, Donovan Newton, Modupe Osinubi, Valentina Osorio, Christine Pearson, Julia Petras, David Philpott, Amy Pickrel, Brandon Potvin, Lalita Priyamvada, Araceli Rey, Erin Ricketts, Sergio Rodriguez, Julie Rushmore, Panayampalli S. Satheshkumar, Hannah Segaloff, Ahlia Sekkarie, Artee Sharma, Emily Sims, Dallas Smith, Teresa Smith, Todd Smith, Dipesh Solanky, Ian Spiknall, Danielle R. Stanek, Mark Stenger, Frank Strona, Kara Tardivel, Eishita Tyagi, Pascale Wortley, Diana Valencia, Michelle Waltenburg, Erin Whitehouse, Marcia Wong

**Affiliations:** ^1^Epidemic Intelligence Service, CDC; ^2^Division of High Consequence Pathogens and Pathology, National Center for Emerging and Zoonotic Infectious Diseases, CDC; ^3^Division of STD Prevention, National Center for HIV, Viral Hepatitis, STD, and TB Prevention, CDC; ^4^Massachusetts Department of Public Health; ^5^New York City Department of Health and Mental Hygiene, New York, New York; ^6^Salt Lake County Health Department, Salt Lake City, Utah; ^7^Florida Department of Health; ^8^Fairfax County Health Department, Fairfax, Virginia; ^9^Public Health - Seattle & King County, Seattle, Washington; ^10^Colorado Department of Public Health and Environment; ^11^Massachusetts General Hospital, Boston Massachusetts; ^12^Georgia Department of Health; ^13^Division of Healthcare Quality Promotion, National Center for Emerging and Zoonotic Infectious Diseases, CDC; ^14^Division of Global Migration and Quarantine, National Center of Emerging Zoonotic Infectious Diseases, CDC; ^15^National Institute for Occupational Safety and Health; ^16^Division of Global Health Protection, Center for Global Health, CDC; ^17^Division of HIV Prevention, National Center for HIV, Viral Hepatitis, STD, and TB Prevention, CDC.; CDC; CDC; CDC; CDC; CDC; CDC; CDC; CDC; CDC; Massachusetts Department of Health; Colorado Department of Public Health and Environment; CDC; CDC; CDC; CDC; CDC; CDC; Fairfax County Health Department; CDC; CDC; CDC; CDC; CDC; Public Health - Seattle & King County; CDC; CDC; CDC; CDC; CDC; CDC; CDC; CDC; CDC; CDC; CDC; CDC; CDC; CDC; CDC; CDC; CDC; CDC; CDC; CDC; CDC; CDC; CDC; CDC; CDC; CDC; CDC; CDC; Utah Department of Health; CDC; CDC; CDC; CDC; California Department of Public Health; CDC; CDC; CDC; CDC; CDC; CDC; CDC; CDC; CDC; CDC; CDC; CDC; CDC; CDC; CDC; CDC; CDC; CDC; CDC; CDC; CDC; CDC; CDC; CDC; Florida Department of Health; CDC; CDC; CDC; CDC; Georgia Department of Public Health; CDC; CDC; CDC; New York City Department of Health and Mental Hygiene.

On May 17, 2022, the Massachusetts Department of Public Health (MDPH) Laboratory Response Network (LRN) laboratory confirmed the presence of orthopoxvirus DNA via real-time polymerase chain reaction (PCR) from lesion swabs obtained from a Massachusetts resident. Orthopoxviruses include *Monkeypox virus*, the causative agent of monkeypox. Subsequent real-time PCR testing at CDC on May 18 confirmed that the patient was infected with the West African clade of *Monkeypox virus*. Since then, confirmed cases* have been reported by nine states. In addition, 28 countries and territories,^†^ none of which has endemic monkeypox, have reported laboratory-confirmed cases. On May 17, CDC, in coordination with state and local jurisdictions, initiated an emergency response to identify, monitor, and investigate additional monkeypox cases in the United States. This response has included releasing a Health Alert Network (HAN) Health Advisory, developing interim public health and clinical recommendations, releasing guidance for LRN testing, hosting clinician and public health partner outreach calls, disseminating health communication messages to the public, developing protocols for use and release of medical countermeasures, and facilitating delivery of vaccine postexposure prophylaxis (PEP) and antivirals that have been stockpiled by the U.S. government for preparedness and response purposes. On May 19, a call center was established to provide guidance to states for the evaluation of possible cases of monkeypox, including recommendations for clinical diagnosis and orthopoxvirus testing. The call center also gathers information about possible cases to identify interjurisdictional linkages. As of May 31, this investigation has identified 17^§^ cases in the United States; most cases (16) were diagnosed in persons who identify as gay, bisexual, or men who have sex with men (MSM). Ongoing investigation suggests person-to-person community transmission, and CDC urges health departments, clinicians, and the public to remain vigilant, institute appropriate infection prevention and control measures, and notify public health authorities of suspected cases to reduce disease spread. Public health authorities are identifying cases and conducting investigations to determine possible sources and prevent further spread. This activity was reviewed by CDC and conducted consistent with applicable federal law and CDC policy.^¶^

Monkeypox, a zoonotic disease for which the animal reservoir is unknown (*1*), is endemic in several Central and West African countries. There are two clades of *Monkeypox virus*, West African, and Congo Basin, the latter causing more severe illness (*1*,*2*). The last United States monkeypox outbreak was secondary to imported small mammals from Ghana in 2003**; however, since monkeypox reemerged in Nigeria in 2017, isolated cases outside Africa have been reported either among persons with recent travel to Nigeria or among secondary contacts of persons with travel-associated cases (*2*,*3*). Patients with monkeypox typically experience a febrile prodrome 5–13 days after exposure (range = 4–17 days), which often includes lymphadenopathy, malaise, headache, and muscle aches; this prodrome might depend on the nature of exposure (*4*). The prodrome is followed 1–4 days later by the onset of a characteristic deep-seated, vesicular or pustular skin rash with a centrifugal distribution (Figure); the lesions are well circumscribed and often umbilicate or become confluent, progressing over time to scabs. The rash can be disseminated. Some recent cases have begun atypically, with lesions in the genital and perianal region and without subjective fever or other prodromal symptoms. For this reason, cases might be confused with more commonly seen infections such as varicella zoster or sexually transmitted infections (STIs) (e.g., genital herpes or syphilis). The case-fatality ratio for the West African clade of monkeypox is reported to be 1% and might be higher in immunocompromised persons (*1*,*5*,*6*).

**FIGURE:**
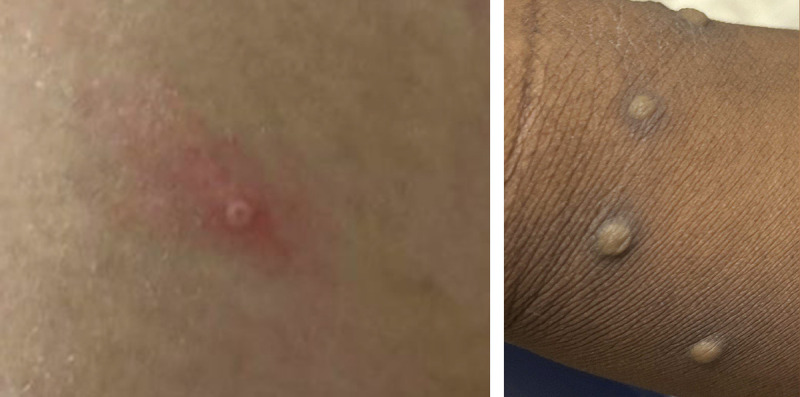
Characteristic monkeypox lesions*^,^^†^ — United States, May 2022 * The rash associated with monkeypox involves firm, deep-seated, and well-circumscribed vesicles or pustules, which might umbilicate or become confluent. Lesions progress over time to scabs. ^†^ Photos used with patients’ permission.

A person is considered infectious from the onset of illness until all lesions have crusted over, those crusts have separated, and a fresh layer of healthy skin has formed under the crust. Human-to-human transmission occurs by direct contact with infected body fluids or lesions, via infectious fomites, or through respiratory secretions, that typically require prolonged interaction (*1*). Historically, documented reports of human-to-human transmission have been among household contacts and shared housing inhabitants (e.g., in prisons), and health care providers who have had close, sustained contact with a patient or patient fomites (e.g., bedding) (*6*,*7*).

## Investigation and Results

**United Kingdom**. The United Kingdom Health Security Agency (UKHSA) announced a confirmed monkeypox case on May 7, 2022, in a traveler returning from Nigeria. On May 14 and 16, UKHSA announced a second unrelated cluster of two cases and a third clustered group of four cases identified at sexual health clinics; the four-case cluster involved persons who identify as gay, bisexual, or MSM.

**Massachusetts**. On May 4, a Massachusetts resident developed an anogenital rash 3 days after returning from international travel. This rash progressed to vesicles and pustules and spread to the face and trunk; the patient sought medical care four times at outpatient clinics during May 4–12, during which time common causes were ruled out. The patient was hospitalized on May 12 for management of refractory perianal pain from the rash. Prompted by UKHSA’s announcement regarding the recent monkeypox cases, clinicians notified the MDPH and CDC for testing. On May 17, the patient received a diagnosis of confirmed *Orthopoxvirus* by the Massachusetts LRN laboratory, and CDC confirmed *Monkeypox virus* West African clade the following day. The local hospital infection prevention team, MDPH, and CDC responded to identify contacts and determine exposure risk, facilitate PEP with one of two orthopoxvirus vaccines (ACAM2000^††^ or JYNNEOS^§§^), and provide guidance on infection prevention and control. Outbreak case definitions were created (Table 1). Exposure risk assessment tools used during investigation of a 2021 travel-associated monkeypox case in Texas (*8*) were adapted to monitor cases and determine criteria for recommending PEP.

**TABLE 1:** Interim clinical, laboratory and epidemiologic criteria for case classification — U.S. Monkeypox Response, May 2022

Clinical and laboratory classification	Criteria
Suspected	New characteristic rash* **OR**
	Meets one of the epidemiologic criteria and has high clinical suspicion^†^ for monkeypox
Probable	No suspicion of other recent orthopoxvirus exposure (e.g., *Vaccinia virus* in ACAM2000 vaccination) **AND** demonstration of the presence of
	• Orthopoxvirus DNA by polymerase chain reaction testing of a clinical specimen **OR **
	• *Orthopoxvirus* using immunohistochemical or electron microscopy testing methods **OR**
	• Detectable levels of antiorthopoxvirus IgM antibody during the period of 4–56 days after rash onset
Confirmed	Demonstration of the presence of *Monkeypox virus* DNA by polymerase chain reaction testing or next-generation sequencing of a clinical specimen **OR**
	Isolation of *Monkeypox virus* in culture from a clinical specimen
**Epidemiologic classification**	
Within 21 days of illness onset	Reports having contact with a person or persons with a similar appearing rash or received a diagnosis of confirmed or probable monkeypox **OR**
	Had close or intimate in-person contact with persons in a social network experiencing monkeypox activity, including MSM who meet partners through an online website, digital app, or social event (e.g., a bar or party) **OR**
	Traveled outside the United States to a country with confirmed cases of monkeypox or where *Monkeypox virus* is endemic **OR**
	Had contact with a dead or live wild animal or exotic pet that is an African endemic species, or used a product derived from such animals (e.g., game meat, creams, lotions, or powders)
**Exclusions**	
A case might be excluded as a suspect, probable or confirmed case if:	An alternative diagnosis* can fully explain the illness **OR**
	A person with symptoms consistent with monkeypox does not develop a rash within 5 days of illness onset **OR**
	A case where high-quality specimens do not demonstrate the presence of *Orthopoxvirus* or *Monkeypox virus* or antibodies to *Orthopoxvirus*

**Abbreviations**: IgM = immunoglobulin M; MSM = men who have sex with men.

* The characteristic rash associated with monkeypox lesions involve the following: deep-seated and well-circumscribed lesions, often with central umbilication; and lesion progression through specific sequential stages: macules, papules, vesicles, pustules, and scabs. The rash can sometimes be confused with other diseases that are more commonly encountered in clinical practice (e.g., secondary syphilis, herpes, and varicella zoster). Historically, sporadic accounts of patients co-infected with *Monkeypox virus* and other infectious agents (e.g., varicella zoster, or syphilis) have been reported, therefore patients with a characteristic rash should be considered to receive testing, even if other test results are positive.

^†^ Clinical suspicion can exist if initial signs and symptoms are consistent with illnesses confused with monkeypox (e.g., secondary syphilis, herpes, and varicella zoster).

**New York**. On May 4, a traveler returning to New York City (NYC) was evaluated for an oral lesion, and a new painful, perianal rash; the patient was tested and treated for a presumed common STI and sent home. The rash spread, progressing to pustules, and the patient was seen again and treated for a different STI; all testing results were ultimately negative. On May 19, after the announcement of the monkeypox case in Massachusetts, a clinician caring for the NYC patient notified the NYC Department of Health and Mental Hygiene (NYC DOHMH) about the possibility of monkeypox. The patient received a positive orthopoxvirus test result at the NYC LRN laboratory and continued to isolate at home. NYC DOHMH began identifying contacts, determining exposure risk, and facilitating PEP for at-risk contacts.

**Other U.S. states**. Over the next 5 days from the identification of the NYC case, multiple states received notifications from clinicians about suspected monkeypox cases; on May 23, an incident command structure was created within CDC’s National Center for Emerging and Zoonotic Infectious Diseases to respond to this outbreak. As of May 31, nine states (California, Colorado, Florida, Georgia, Massachusetts, New York, Utah, Virginia, and Washington) have reported 17 patients with confirmed orthopoxvirus infections, which until proven otherwise, are considered to be *Monkeypox virus* during this outbreak response (Supplementary Figure 1, https://stacks.cdc.gov/view/cdc/117901).

Fourteen patients of the 17 patients reported international travel involving 11 different countries during the 21 days preceding symptom onset, and 16 of the 17 patients identified as MSM. All patients were adults (average age = 40 years; range = 28–61 years), and all had rash onset dates during May 1–27; three patients were immunocompromised. Diagnosis of an orthopoxvirus infection occurred an average of 11 days after rash onset (range = 0–21 days) (Supplementary Figure 2, https://stacks.cdc.gov/view/cdc/117900). In addition to skin rash, patients commonly reported chills (12), fatigue or malaise (11), and lymphadenopathy (nine); fever was reported in seven patients (Table 2). Twelve patients reported prodromal symptoms before rash onset such as fatigue, fever, or headache. Among eight patients, the rash started in the genital or perianal area. All but one patient developed a disseminated rash, occurring on the arms, trunk, legs, and face.

**TABLE 2:** Clinical characteristics of patients with confirmed orthopoxvirus and monkeypox (N = 17) — United States, May 2022*

Characteristic	No. (%)		
	At illness onset	Prodromal period^†^	At any point in illness
**Signs and symptoms^§^ during illness**			
Rash	5 (29)	NA	17 (100)
Fatigue or malaise	3 (18)	13 (76)	13 (76)
Chills	0 (—)	4 (24)	12 (71)
Lymphadenopathy	0 (—)	1 (6)	9 (53)
Inguinal	0 (—)	0 (—)	6 (35)
Cervical^¶^	0 (—)	1 (6)	3 (18)
Headache	2 (12)	5 (29)	8 (47)
Fever	6 (35)	5 (29)	7 (41)
Body ache	1 (6)	2 (12)	6 (35)
Sore throat or cough	2 (12)	3 (18)	5 (29)
Sweat	1 (6)	2 (12)	4 (24)
Other	3 (18)	4 (24)	13 (76)
**Rash locations** ^§^			
Arm	4 (24)	NA	9 (53)
Trunk	1 (6)	NA	9 (53)
Leg	0 (—)	NA	8 (47)
Face	2 (12)	NA	7 (41)
Hand	1 (6)	NA	6 (35)
Perianal	5 (29)	NA	6 (35)
Oral	0 (—)	NA	5 (29)
Neck	1 (6)	NA	5 (29)
Genital (penis or vagina)	4 (24)	NA	4 (24)
Feet	1 (6)	NA	4 (24)

**Abbreviation**: NA = not applicable.

* Data final through May 31, 2022, 11:59 p.m EDT.

^†^ Any symptoms before rash onset. The development of initial symptoms (e.g., fever, malaise, headache, and weakness) marks the beginning of the prodromal period.

^§^ Multiple response options possible per patient.

^¶^ In one patient it was unclear when cervical lymphadenopathy occurred in relation to rash.

## Public Health Response

Currently, all patients are clinically well and being monitored by health authorities to determine the end of isolation (i.e., after all lesion scabs have fallen off, and new, healed skin has formed). One patient was treated with tecovirimat, an antiviral agent from the strategic national stockpile with antiorthopoxvirus activity, licensed for smallpox but available from CDC under an expanded access Investigational New Drug protocol (*9*). CDC also facilitated the availability of vaccine PEP to contacts with high-risk exposures (e.g., unprotected contact with the skin or mucous membranes, lesion, or body fluids of a patient) or certain intermediate risk exposures (e.g., being within ≤6 ft of an unmasked patient for ≥3 hours without wearing, at a minimum, a surgical mask). PEP is not recommended for low or uncertain risk (e.g., health care providers entering a patient’s room without eye protection). Eligible intermediate- and high-risk contacts are offered PEP with ACAM2000 or JYNNEOS vaccines.

Contact investigation is ongoing; among the 13 patients who have identified contacts, there are 56 high-, 117 intermediate-, and 235 low- or uncertain-risk contacts. Contacts are recommended to be monitored for signs and symptoms consistent with monkeypox (e.g., fever, chills, lymphadenopathy, and rash) for 21 days following last exposure.

Genome sequencing results from virus recovered from the patient in Massachusetts display similarities to other published genomes in this outbreak from Europe (Nextstrain/monkeypox)^¶¶^ and are related to the 2017–2018 monkeypox outbreak in Nigeria. As of June 2, preliminary data indicates approximately 800 monkeypox cases have been reported in this outbreak from 28 countries, including the United States.***

## Discussion

The current identification of monkeypox clusters in several countries that do not have endemic disease and involving patients with no direct travel history to an area with endemic monkeypox suggests person-to-person community spread. Close contact with infected persons or fomites (e.g., shared linens) is the most significant risk factor for *Monkeypox virus* infection in human monkeypox outbreaks (*10*). *Monkeypox virus* is spread through close, often sustained skin-to-skin contact, but the initial appearance or occurrence of lesions in the anogenital area observed in the current outbreak differs from the typical appearance or occurrence beginning on the face, oral mucosa, and hands and feet, then spreading to other parts of the body in a centrifugal distribution. The high proportion of initial cases diagnosed in this outbreak in persons who identify as gay, bisexual, or other MSM, might simply reflect an early introduction of monkeypox into interconnected social networks; this finding might also reflect ascertainment bias because of strong, established relationships between some MSM and clinical providers with robust STI services and broad knowledge of infectious diseases, including uncommon conditions. However, infections are often not confined to certain geographies or population groups; because close physical contact with infected persons can spread monkeypox, any person, irrespective of gender or sexual orientation, can acquire and spread monkeypox.

The following measures can be taken by the public to prevent infection with monkeypox: 1) isolate ill persons from uninfected persons; 2) practice good hand hygiene and use appropriate personal protective equipment to protect household members if ill or caring for ill persons at home (e.g., a surgical mask, long sleeves and pants, and disposable gloves); 3) use an Environmental Protection Agency–registered disinfectant with an emerging viral pathogens claim that is found on EPA’s List Q for disinfection of surfaces.^†††^ Patients should also avoid contact with pets and other animals while infectious, because some mammals might be susceptible to monkeypox. Persons with symptoms of monkeypox, including unexplained lesions, should contact their health care provider for an evaluation and should avoid close contact with others, including intimate or sexual contact, until they are evaluated or receive testing.

CDC urges health care providers in the United States to be alert for patients who have rash illnesses consistent with monkeypox, regardless of a patient’s gender or sexual orientation or a history of international travel or specific risk factors for monkeypox. Clinicians should contact their local or state health department if they suspect a case of monkeypox. There are 110 LRN laboratories available and equipped for rapid diagnostic testing of emerging pathogens across the United States; currently 68 test for orthopoxviruses. The prolonged interval from rash onset to positive test result was reflective of delays in clinical suspicion of an unfamiliar illness; all patients had results within 0–2 days after specimens were collected. During this outbreak, a positive test result for an *Orthopoxvirus* at an LRN laboratory is presumed to be monkeypox and is actionable for antiorthopoxviral treatment, and by public health authorities to initiate isolation, contact tracing, monitoring, investigation, and PEP of exposed contacts. PEP with smallpox vaccines remains available from the strategic national stockpile for eligible exposed persons.

As the source and spread of this outbreak are being investigated, it is crucial to assess all possible modes of transmission and identify risk groups, as well as institute appropriate public health preventive measures. CDC is providing guidance on case definitions, identification of contacts, clinical management, and infection control and prevention within health care facilities and the home, creating resources for disseminating information on monkeypox, and supporting laboratory testing infrastructure domestically and globally.^§§§^

**Summary**

**What is already known about this topic?**

Monkeypox, a rare disease caused by infection with *Monkeypox virus*, is endemic in several Central and West African countries. Cases in persons outside Africa are often linked to international travel or imported animals.

**What is added by this report?**

CDC is tracking multiple reported U.S. monkeypox cases, and monitoring cases in persons in countries without endemic monkeypox and with no known travel links to an endemic area; current epidemiology suggests person-to-person community spread.

**What are the implications for public health practice?**

CDC urges health departments, clinicians, and the public to remain vigilant, institute appropriate infection prevention and control measures, and notify public health authorities of suspected cases to reduce disease spread.

## Footnotes

* Confirmed case for this outbreak is defined as either a positive orthopoxvirus assays or *Monkeypox virus* assays. Case counts as of May 31, 2022 at 11:59 p.m. EDT.

† Argentina, Australia, Austria, Belgium, Canada, Czech Republic, Denmark, Finland, France, Germany, Gibraltar, Hungary, Ireland, Israel, Italy, Malta, Mexico, Morocco, Netherlands, Norway, Portugal, Slovenia, Spain, Sweden, Switzerland, United Arab Emirates, United Kingdom, and United States.

§ One case in Florida was initially identified in another country and is not included in this report.

¶ 45 C.F.R. part 46, 21 C.F.R. part 56; 42 U.S.C. Sect. 241(d); 5 U.S.C. Sect. 552a; 44 U.S.C. Sect. 3501 et seq.

**
https://www.cdc.gov/mmwr/preview/mmwrhtml/mm5223a1.htm

††
https://www.fda.gov/media/75792/download

§§
https://www.fda.gov/media/131078/download

¶¶
https://nextstrain.org/monkeypox

*** Sources include publicly available information from official government or government affiliated websites and verified media information quoting health officials.

†††
https://www.epa.gov/pesticide-registration/disinfectants-emerging-viral-pathogens-evps-list-q

§§§ https://www.cdc.gov/poxvirus/monkeypox/index.html
